# Needs Must When—

**Published:** 1920-09-04

**Authors:** 


### "NEEDS MUST WHEN ——"

By a SOCIAL WORKER.

A LETTER signed "General Practitioner" (August 14) has given us in black and white an opinion which is more generally implied than stated. *For large numbers of the population complete sexual abstinence is impossible, in the literal sense of that word. Human nature being what it is, no amount of propaganda will abolish promiscuous intercourse.* The Christian conflict with the lower impulses is, then, mere folly, victory being impossible. Of no use to found a Round Table, Sir Galahad is born to be what no one can become by trying. It is a dangerous kind of argument, because it may fairly be argued that human nature being what it is, no amount of propaganda will abolish lying, theft, or the amiable habit of putting inconvenient people out of the way. And if these things are so, what right have we to blame the doers of actions which it is for them "impossible" not to do? So modern civilisation may, without self-reproach, "reel back into the beast and be no more." The point I want to make, however, is that this assumption implies the prostitute, and those of us who are working for reconstruction find already that in theory and practice this "mournful and even terrible figure," as Lecky described it, stands sorely in the way.

It is plain that the crushing of the middle classes by taxation and high prices will defer marriage even longer than young men with ideals are apt to defer it to-day—until, that is, they feel able to offer decent conditions and prospects to a girl of their own class. Meantime, like Mr. Kipling's Bessie, Miss Wrong must serve until Miss Right comes along, some mother's daughter must be doomed to the short life, or life prolonged into the hideous old age of procuress or some other forlorn and shady career, of the ministrant to men's confessed yet unacknowledged needs. She must bear the loneliness, the outcast position, the uncertainty of daily bread, the unhappiness painfully kept at bay, perhaps the hidden anguish of the grief and shame of parents, sooner or later ill-health, and the loss of the woman's ordinary happiness and home, pride, and position in her little world.

Hundreds of good women are working in Rescue Societies, spending large sums of money in the aggregate, facing difficulty and disappointment and grudging no pains, so that these poor girls may be put back into family life and happy surroundings. "Fallen" women, they are called, and those who see their misery do not wonder that some vehement feminists have of late years made themselves ridiculous in the eyes of the world by demanding that we should speak also of "fallen" men. The world shrugs its shoulders and observes that from physiological reasons the case of the man is quite different from that of the woman. Then, though the ultra-feminists cannot deny this—and, indeed, have no mind to do so—they counter with an argument which is not so easily disposed of—namely, that if chastity is indeed impossible for men, there are women for whom it is to the same extent impossible. Society in the past has told these that they are not to say "impossible." They, being women, must restrain themselves. If no man offered honourable marriage, there have been for the more educated classes the convent and its vow, its fasts, and its scourge, or the prolonged subserviency of the drawing-room at home and the chill autumn of the wallflower and old maid, the social failure. There are extremists who even venture to claim for the woman of to-day the man's readily conceded right to obey the demands of nature, even if she does not desire to enter the exacting profession of wife and mother. If in so many departments of life it is admitted that sauce for the goose should be as sauce for the gander, why arbitrarily draw, they say, the line at this point? The fact that decent people hardly care to pursue the discussion may be a stronger argument than appears on the surface. Meantime, happily, the energetic "bachelor woman" replaces the forlorn old maid. But, again, the prostitute is a citizen *tout comme une autre*, and national reconstruction must take account of her. In a discussion on her position some time ago some warm-hearted ladies grew eloquent.

"Do not leave her apart," they said; "go and have tea with her. Ask her to tea with you." But the majority of the large audience obviously felt no mind to give opportunity in their own homes for the practising of the oldest trade in the world. The Second Mrs. Tanqueray is not an inviting figure for a new social order; we are left with the paradox that men have needs that must be met, but the women who supply the demand will receive for their wages fine food and clothing, plus shame and contempt from men as well as from women. And for them there is no scheme of insurance against unemployment. Surely this is very unfair, and if we argue that the shame is necessary to mark society's horror of impurity (in women) and to check it, the answer is now openly given that both abstention and early marriage are impossible for a large number of men. The supply must therefore be maintained.

Yet what new-made mother—we can hardly except the most depraved unless she is also half-witted—looking down at a smiling girl-baby in her arms, would willingly be told that its destiny would be the fulfilment of this "necessary" function? This generation declares each child valuable to the nation as an individual citizen. Those of us who study social questions foresee an outcry presently, probably after Christmas when continuation schools come into being, when that of which teachers in our cities are now too well aware becomes matter of common knowledge. This—that a not negligible number of girls sitting in the day at school desks earn liberal pocket-money at night. Things unspeakable are covered by this veil of the community's ignorance. It is only a few years since a lady brought to the face of a child of thirteen, waiting confinement in a charitable Home, the first smile seen there since her entry, by putting a doll into her arms. But it is also with the child of this child that reconstruction has to reckon.

I have known of late years a family, a singularly respectable looking father, a mother, a crippled daughter, and a little grandchild, who largely lived on the earnings of the remaining daughter, a night-walker whose trade was of no very high order. Incidentally she had inveigled into marriage a young Canadian soldier, who had learnt the truth and left her. The child—of which daughter was uncertain—was a pretty child, but always suffering from sores. If she lives to grow up, what hope is there that she will not pass on a disease in practising vice? This is just a stray picture from the tragic book of fate of the necessary ministrant to a declared need. Is it not time that we drive this problem into open daylight and ask whether another "impossible" must not also come into being and the status of this servant of the community be recognised and respected? There are only two alternatives, the clear teaching to boys and acceptance by young men of self-control as a duty, to which should be added a popularising of early marriage for those who need it, women as well as men, and this frank acceptance of the prostitute as public servant. To say that "as it was in the beginning it now is and ever will be" is no answer, because for the first time civilisation is making a definite attempt which it calls Reconstruction to set its house in order. And this question waits for it on the threshold, an insistent creditor holding an age-long account, which can no longer be shirked. Over his shoulder looks the figure of Religion, and adds, "Is it not Civitas Dei that you promise to build?"

**[See letter from "General Practitioner," p. 576.]**

